# Fibre Composition and Maturity of Forage-Based Diets Affects the Fluid Balance, Faecal Water-Holding Capacity and Microbial Ecosystem in French Trotters

**DOI:** 10.3390/ani13030328

**Published:** 2023-01-17

**Authors:** Sara Muhonen, Véronique Julliand

**Affiliations:** L’institut Agro Dijon, Burgundy Franche-Comté University, PAM UMR A 02.102, 21000 Dijon, France

**Keywords:** equine, faecal microbiota, haylage, lucerne

## Abstract

**Simple Summary:**

Horses are herbivores and hindgut fermenters, which makes forage-based diets a natural choice. Traditionally, diets for horses have comprised large portions of starch-rich concentrate shown to adversely affect intestinal health. Feeding more forage and less concentrate benefits health and performance in horses. However, forage nutritional quality can differ greatly and further studies on forage diets for horses are needed. Increased knowledge on which type of forages are suitable for different horse categories is important for the horse industry, advisors, veterinarians and the diet formulations industry. This study compared a grass forage diet, a legume forage diet and a concentrate and forage diet, on the fluid balance, body weight and function of the large intestine in French trotters. The results showed that an early-harvested grass forage diet implied greater faecal water-holding capacity without increasing body weight and greater faecal concentrations of total bacteria. In conclusion, early-harvested forage might promote the fluid balance of high-performing horses. Early-harvested forage that can fulfil the energy and protein requirements with no need to add concentrate also promotes hindgut health.

**Abstract:**

Racing events challenge the fluid balance of athletic horses. The equine large intestine functions as a fluid reservoir, since the properties of dietary forage fibre affect the digesta water content and the milieu of this ecosystem. This study aimed to investigate the effect of grass maturity and legume forage on the faecal water-holding capacity (WHC) and microbial ecosystem, and the fluid balance and body weight (BW) of French trotters in race training. Six geldings were offered three diets with different fibre compositions: concentrate and late-harvested mature grass haylage (35:65 energy ratio) (CMGH); early-harvested grass haylage and mature grass haylage (80:20) (EGH); and lucerne and mature grass haylage (80:20) (LH), for 24 days in a Latin square design. Body weights were lower and faecal WHC higher when the horses were fed EGH compared to CMGH and LH (485 vs. 492 and 492 kg, *p* < 0.001; 12.6 vs. 11.1 and 11.4 g H_2_O/g dry faeces, *p* = 0.014, respectively). Total water intake and output did not differ between diets, but water excretion via faeces was lower and via urine was greater on EGH compared to CMGH and LH (13.1 vs. 18.8 and 17.6 kg, *p* = 0.001; 10.5 vs. 7.6 and 7.9 kg, *p* = 0.032, respectively). Total bacteria concentrations were higher on EGH than CMGH and LH (5.4 × 10^11^ vs. 2.8 × 10^11^ and 2.8 × 10^11^ CFU/mL, *p* = 0.018, respectively). Concentrations of butyrate were greater, and pH was lower when fed EGH compared to CMGH and LH (3.0 vs. 2.0 and 1.6 mmol/L, *p* = 0.034; 6.5 vs. 6.9 and 7.2, *p* = 0.005, respectively). In conclusion, forage harvested at an early stage of maturity could benefit athletic horses’ fluid balance by providing a more available large intestine fluid reservoir without increasing BW.

## 1. Introduction

Horses are herbivores and hindgut fermenters and, therefore, have a minimum requirement of fibre in their diet. Too low a forage intake can adversely affect health, welfare, behaviour and performance in horses [1,2,3]. As more plant-fibre and forage-based diets are recommended for athletic horses, new questions are raised concerning which type of forage is the most suitable.

Racing events challenge the fluid balance of athletic horses, and the equine large intestine may act as a fluid reservoir, as fibre holds water in the digesta [4,5,6]. Due to this large fluid and electrolyte reservoir in the large intestine, high fibre diets can be covetable for athletic horses [7]. In addition, recent advances in equine nutrition recommend more forage-based diets for horses, as they benefit gut health [1]. However, the physical characteristics and chemical composition of forages influences not only health aspects [8], but also gut fill and body weight (BW) [9]. An increased BW may be a disadvantage for high-performing horses [10], but BW changes appear to depend more on the type of forage than actual forage DM intake [11,12]. Using sedentary maintenance fed horses, an early-harvested grass haylage diet resulted in a higher water-holding capacity (WHC) of the caecum and right ventral colon digesta, but not in heavier horses, compared to a legume haylage diet and a late-harvested grass haylage and concentrate diet [13]. 

The WHC of fibre depends on both the maturity and variety of the fibre [14]. How dietary fibres function in the large intestine rely on the proportion of microbial digestion and, therefore, fibrolytic microorganisms play an important role for the WHC [15]. Fibres with high WHC ferment to a greater extent [16], and water is made available as the fibre is being fermented by the intestinal microbiota [17]. A higher inclusion of fermentable fibre in the diet may, thus, provide a greater volume of available fluid in the large intestine. 

Both early and more recent studies have shown that grass development and structural change influence the digestibility of dietary components [18,19]. The nutritional value of forage decreases as the cell wall content increases with an advancing stage of maturity [19]. Furthermore, legumes and grasses differ in both fibre digestibility and the concentration of fibre. Legumes generally comprise less fibre, less-digestible fibre and more lignified fibre compared to grasses. Fibre quality and quantity have a great influence on the enzymatic activity of cellulolytic bacteria and, therefore, short-chain fatty acid (SCFA) production [20,21,22]. Legume forages such as lucerne have a high concentration of pectin, and pectin is persistently the most rapidly degraded complex carbohydrate, compared to the degradation rates of celluloses and starches that are rather variable [23]. Rumen degradation kinetics have shown large differences between legumes and grasses [24]. 

Comparisons between athletic and sedentary horses fed the same diets resulted in more distinct differences for the athletic horses for measurements such as water intake, faecal DM and pH, and plasma urea [25]. This is probably because high-performing horses are fed about twice the maintenance level. Therefore, to investigate the effects on faecal WHC and microbiota, BW and fluid balance using exercising horses may contribute crucial knowledge to previous studies using fistulated horses. 

The aim of this study was to investigate the effect of feeding early-harvested grass haylage and lucerne haylage, compared to the more conventional concentrate and mature grass haylage diet on the faecal WHC and microbiota, and the fluid balance and BW of French trotters in race training.

## 2. Materials and Methods

The experiment was conducted under the ethical committee of the Burgundy University (agreement no. B0810).

### 2.1. Animals, Design and Training 

Six French trotters (geldings, aged 3 to 7 years) in race training were used. The horses’ BW ranged from 448 to 543 kg. Horses were kept in single boxes on wood shavings and spent 1–3 h daily in sand paddocks. The animals were dewormed with Eqvalan Duo (Merial, France) nine weeks before the experiment started. All the horses were randomly assigned to three diets in a Latin square design. Each experimental period was 24 days, and during the first five days the diets were gradually changed. During each experimental period the horses performed six training sessions 2–4 days apart: (1) submaximal exercise: 6000 m slow trot (pace 6–7 m/s); (2) intensive interval exercise: 3000 m slow trot, 2 × 1125 m with heart rates (HR) >200 beats/min; (3) submaximal exercise: 8000 m slow trot (pace 6–7 m/s); (4) intensive interval exercise: 3000 m slow trot, 3 × 1125 m with HR >200 beats/min; (5) intensive interval exercise: 3000 m slow trot, 2 × 1125 m with HR >200 beats/min; and (6) intensive interval exercise: 3000 m slow trot, 4 × 1125 m with HR >200 beats/min.

### 2.2. Diets

The experimental forages were two grass haylages, first cuts from the same field but harvested at different stages of maturity, and a lucerne haylage. One half of the grass ley was harvested as the early grass haylage and cut on the 31 of May, and the other half harvested as the mature grass haylage and cut on the 13 of July (Lat. 59° N., Long. 17° E.). The two haylages harvested in different stages of maturity resulted in in vitro digestible organic matter (IVDOM) values of 87% for the early-cut and 64% for the late-cut mature grass haylage. In addition, the different stages of maturity were reflected in metabolisable energy (ME) and chemical composition: 11.3 MJ, 175 g of CP, 288 g of crude fibre, 536 g of NDF, 309 g of ADF, 28 g of ADL per kg DM, and 7.5 MJ, 91 g of CP, 381 g of crude fibre, 656 g of NDF, 412 g of ADF, 52 g of ADL per kg DM for the early and the mature grass haylage, respectively. The grass forages were primarily timothy and ryegrass. The lucerne (*Medicago sativa*) forage was harvested at early bloom on the 11 of August (Lat. 47° N., Long. 7° E.) (second cut and weed inclusion estimated to be about 10%). The IVDOM of the lucerne was 66% and the chemical composition: 8.8 MJ, 144 g of CP, 355 g of crude fibre, 508 g of NDF, 402 g of ADF and 75 g of ADL per kg DM.

To obtain similar intakes of ME and CP, the diets were fed restrictedly: the conventional diet of concentrate and mature grass haylage in a 35:65 energy ratio, i.e., 35% of the energy intake was provided by concentrate (rolled oats, soybean meal) and 65% was provided by the forage (CMGH); the grass diet with early and mature grass haylage in an 80:20 energy ratio (+ small amount soybean meal) (EGH) and the legume diet with lucerne haylage and mature grass haylage in an 80:20 energy ratio (LH) (Table 1). Individual diets were calculated to fill the energy (0.5 MJ ME × bwt^0.75^), mineral and electrolyte requirements [26]. All diets were supplemented (65 g/day) with a commercial mineral product (Krafft, Falkenberg, Sweden) and salt (40 to 45 g/day). Additional chalk (calcium carbonate) was added to the CMGH and EGH to assure an isocalcium intake. The horses were fed approximately 20% of the daily feed allowance at 08.00 h, 12.00 h and 16.00 h, and 40% at 18.30 h. At each meal, the same ratios for concentrate:forage and forage:forage were fed.

### 2.3. Feed, Faeces, Urine and Blood Sampling and Measures

A 72 h total collection of urine and faeces was performed at the end of each experimental period using collection harnesses permitting a separate collection of faeces and urine. The harnesses were monitored on an hourly basis, with urine bags emptied continuously and faeces every 12 h. Urine and faecal samples were prepared and analysed for DM as previously described [13]. The horses were weighed at 08.30 h three times weekly and daily during the 72 h collection. The animals had access to water ad libitum, and the water intake was measured during the 72 h faecal and urine total collection. At the end of the experimental periods, the horses’ body condition score (BCS) was assessed by a person not knowing which diet the horses were fed, according to the BCS method of INRA-HN-IE, using a scale of 0–5 [28].

Samples of feedstuffs for the chemical analyses were obtained daily during the 72 h collection period of faeces and urine. The feed samples were prepared and analysed for DM, ash, CP, crude fat, gross energy, crude fibre, NDF, ADF, ADL, starch, water soluble carbohydrates, IVDOM and minerals, as previously described [29].

Faecal grab-samples were taken from the rectum at 12.30 h at the end of each experimental period for classic and molecular microbial analyses, SCFA and pH, and prepared and analysed as previously described [29]. The faecal grab-samples were also prepared and analysed for DM and WHC as previously described [13].

At 12.00 h, at the end of the experimental periods, blood was sampled by venipuncture from the jugular vein into heparinized tubes, centrifuged (1500× *g*, 10 min) and frozen (−20 °C). Total plasma proteins (TPP) were measured with a refractometer (Rogo-Sampaic, Wissous, France).

### 2.4. Statistical Analyses

Before the statistical analysis, logarithmic transformations (log_10_) were carried out on colony counts for concentrations of cultured bacteria. An analysis of variance was performed utilising the SAS software PROC MIXED (SAS Inst., Inc., Cary, NC). All variables were analysed using a statistical model including fixed (period, diet) and random (horse) effects. The model elements were the overall mean, the effect of horse, the effect of period, the effect of diet, the effect of interaction between period and diet, and the random error. The main effect means were separated using pair-wise t-tests. Values are shown as the least square means of six horses with the pooled standard error of the mean (SEM). Differences were considered statistically significant at *p* < 0.05. The PROC CORR of the SAS software (SAS Inst., Inc., Cary, NC) was used to calculate Pearson correlations (r), and *p*-values were calculated for r = 0.

## 3. Results

### 3.1. Feed Intake

Feed intakes are shown in Table 1. When the horses were fed CMGH, two horses refused on average 0.2 kg of DM/day of the grass haylage, and when fed EGH, one horse refused on average 0.6 kg of DM/day of the mature grass haylage. When the horses were fed LH, all horses refused on average 1.7 kg of DM lucerne haylage/day, except for one horse that instead gained weight, and his daily ration had to be decreased by 1.4 kg of DM lucerne haylage and 0.4 kg of DM grass haylage. Body weights were lower when the horses were fed EGH than when they were fed CMGH and LH (CMGH: 492 kg, EGH: 485 kg, LH: 492 kg, SEM = 14.5, *p* < 0.001). The horses’ BCS did not differ between diets (CMGH: 3.2, EGH: 3.4, LH: 3.4, SEM = 0.19, *p* = 0.053).

### 3.2. Fluid Balance and Faecal Water-Holding Capacity

The horses drank more when they were fed CMGH and EGH than LH, but there was no difference in total water intake (drinking + water in feed) between the diets (Table 2). The ratio of total water intake over total DM intake was greater when the horses were fed EGH and LH than CMGH (CMGH: 3.2, EGH: 3.6, LH: 3.6, SEM = 0.07, *p* = 0.005). Total water intake was correlated to total DM, NDF and ADF intake, and to total forage DM, NDF and ADF intake (Table 3). The daily excretion of water via faeces was lower and the excretion of water via urine was higher when the horses were fed EGH compared to CMGH and LH (Table 2). Total water output (water in faeces + water in urine) and the difference between total water intake and water output did not differ between diets (Table 2). The resting values of TPP did not differ between diets (CMGH: 62.2 g/L, EGH: 62.5 g/L, LH: 62.0 g/L, SEM = 0.43, *p* = 0.647). Measured with the centrifugation method, the faecal WHC was greater when the horses were fed EGH than CMGH and LH (Table 4). There were no differences between diets for faecal DM concentrations and the faecal WHC measured with the filtration method (Table 4). Faecal DM concentration and faecal WHC were not correlated (data not shown).

### 3.3. Faecal Bacterial Flora, Short-Chain Fatty Acids and pH

**Figure 1:**
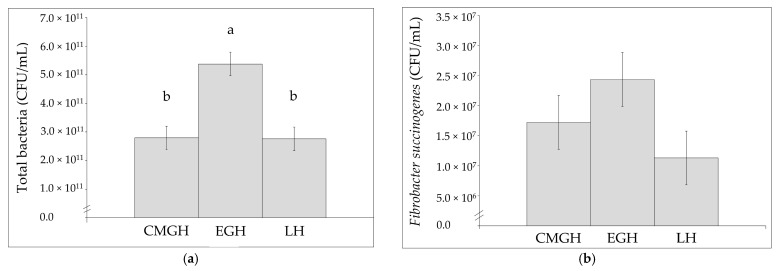
Concentrations of (**a**) total bacteria and (**b**) *Fibrobacter succinogenes* in faeces after three weeks of adaptation to forage-based diets differing in fibre composition and maturity. Concentrations of total bacteria were greater when the horses were fed EGH compared to CMGH and LH (*p* = 0.018). Values are least square means (with the pooled SEM) (*n* 6). ^a,b^ Means without a common superscript letter differ (*p* < 0.05). CMGH: concentrate (oats, soybean meal) and mature grass haylage (35:65 energy ratio), EGH: early and mature grass haylage (80:20) (+ small amount of soybean meal), LH: lucerne haylage and mature grass haylage (80:20).

Using the culturing technique, total viable anaerobic bacteria, pectinolytic, xylanolytic, cellulolytic and amylolytic bacteria concentrations did not imply any differences between the diets (Table 5). Concentrations of lactate-utilising bacteria were greater when the horses were fed CMGH compared to EGH and LH (Table 5). Using the real-time qPCR analysis, the absolute values of total bacteria were greater when the horses were fed EGH compared to CMGH and LH (Figure 1a). Concentrations of *F. succinogenes* did not differ between diets (Figure 1b). The presence of *R. flavefaciens* and *R. albus* was not detected. There were no differences between diets for concentrations of total SCFA, acetate, propionate, valerate, iso-butyrate, iso-valerate, D-lactate, L-lactate and the SCFA ratio (acetate + butyrate/propionate) (Table 5). Butyrate concentrations were greater, and pH was lower on EGH than CMGH and LH (Table 5).

## 4. Discussion

The objective of this study was to examine the differences in fibre composition and maturity of forage-based diets on the faecal WHC and microbiota, and fluid balance and BW of exercising horses. The decrease in IVDOM from 87% to 64% reflects the increase in the stage of maturity of the grasses. The diets resulted in different fibre intakes; EGH provided a lower and LH a greater intake of ADF, ADL and crude fibre, EGH lower cellulose intake and LH lower hemicellulose intake, whereas CMGH supplied a greater intake of NDF. The energy intake was lower (2.4–3.2 MJ/100 kg BW per day) when the horses were fed LH compared to CMGH and EGH. The lower energy intake on LH was due to refusals in five horses and restriction of the ration for one horse. This one horse gained 13 kg in the first nine days with LH. Therefore, his diet was restricted with the same amount as the refusals of the other horses when they were fed LH (200–300 g/100 kg of BW per day). Despite the lower energy intake when LH was fed, the horses did not lose BW. It is known from practice that lucerne is effective for fattening horses.

The horses’ BWs were lower when fed EGH compared to LH and CMGH, which might be explained by the lower intake of less-digestible fibre, resulting in less mass of undigested organic matter and, in total, less water in the large intestine [12]. In contrast, EGH resulted in greater faecal WHC than CMGH and LH. Similar results were obtained in a previous study using sedentary fistulated horses [13]. This would mean that greater WHC of the hindgut digesta does not necessarily imply a greater BW of the horse. This is consistent with a previous study [30] that showed an inverse relationship between the WHC of different fibres and their influence on the faecal bulking of water, indicating that dietary fibre does not influence faecal weight merely by retaining water in the intestine.

In vitro studies have reported that fibres holding more water also ferment to a greater extent, and fermentation instantly reduces the fibre’s WHC, but instead, the addition of microbial mass contributes to the WHC of the intestinal content [16,31]. Sugars also influence microbial mass, but probably earlier in the gastrointestinal tract of equines [32]. The greater concentration of faecal total bacteria might have contributed to the greater faecal WHC on EGH due to the addition of microbial OM [16]. The higher concentration of faecal total bacteria, faecal butyrate and lower faecal pH indicated a more extensive hindgut fermentation when the horses were fed EGH than CMGH and LH. EGH also resulted in lower water excretion via faeces, and in combination with greater faecal WHC this might suggest a greater availability of water in the large intestine. This would be in accordance with the significant relation between microbial digestion and the net movement of Na and water across the intestinal mucosa [33]. The large difference in faecal water excretion and lack of difference in faecal DM also suggests a mechanical effect as the colonic separation mechanism assures the retention of water or preferential transfer of DM at the boundary between the dorsal colon and distal colon [34,35].

TPP did not differ between the diets, suggesting that there was no effect on the plasma volume. However, blood samples were drawn four hours after feeding, and the post-feeding rise in TPP [12,36] may have concealed potential diet effects. Lower TPP was reported when a forage-only diet was compared to a forage-oats diet 5 to 12 h after fasting, indicating a better-maintained plasma volume during fasting for the forage-only diet [12]. A diet effect on TPP concentration during exercise was shown with lower TPP when horses were fed an ad libitum hay diet, indicating a larger movement of water from the gastrointestinal tract to the plasma volume compared to a limited hay diet [8].

Water intake in horses has been reported to correlate with DM intake and the diet cell-wall constituents [37]. In this study, the total water intake correlated to total ration DM, NDF and ADF intake, and it also correlated to forage DM, NDF and ADF intake. This contrasts with sedentary horses fed the same three diets at maintenance level, where total water intake did not correlate to total ration DM, NDF or ADF intake, but did correlate to forage DM and forage ADF intake [13]. The water-to-feed ratio was also higher for EGH and LH than CMGH, which corresponds to previous results with all forage diets resulting in a greater water-to-feed ratio compared to hay-grain diets [37].

Although a comparatively small daily starch intake (<2 g/kg BW) on CMGH, concentrations of lactate-utilising bacteria were greater on CMGH than EGH and LH, but amylolytic bacteria did not differ. However, there were no differences in lactate concentrations between the diets, indicating that the increase in lactate-utilising bacteria moderated a possible increase in lactate. That quite a small daily starch intake as <2 g/kg BW appeared to influence faecal lactate-utilising bacteria is disturbing. Feeding the same diets at maintenance level with a daily starch intake of <1 g/kg BW to fistulated horses also resulted in higher lactate-utilising bacteria concentrations, as well as higher L-lactate concentrations in the caecum and colon for the concentrate and mature grass haylage diet [29].

The greater faecal butyrate concentration on EGH needs further investigation. The butyrate concentration in caecal and colonic fluid has been reported to be approximately 6% of total SCFA [3]. The faecal butyrate concentration when the horses were fed EGH was 6% of total SCFA. When the same three diets were fed at maintenance level to sedentary fistulated horses, the butyrate concentration in the caecum and right ventral colon was 8% but did not differ between diets. Butyrate produced by microbial fermentation in the large intestine is a major metabolite for the colonic epithelial cells and a cellular mediator regulating multiple functions of gut cells and more [38]. Human colonic butyrate producing bacteria are Gram-positive firmicutes and have been shown to play a decisive role in colon health in humans, primarily by producing butyrate [39,40]. Butyrate as a dietary supplement has also been tested in healthy horses [41]. It would be of great interest to explore the influence and importance of butyrate on the equine hindgut mucosa.

## 5. Conclusions

The limitations of this study include a lack of data from the caecum and colon, which instead, were obtained in previous studies using fistulated horses [13,29]; conversely, fistulated horses cannot be exercised as non-fistulated horses. In forage-feeding studies, it is difficult to ensure exact amounts of intake of different nutrients, and in feeding experiments a larger number of experimental horses is always desirable.

A forage-only diet does not necessarily imply higher WHC of hindgut digesta, and higher WHC of the hindgut digesta does not necessarily imply a greater BW of the horse. Forage harvested at an early stage of maturity may imply an advantage for the fluid balance of high-performing horses, rendering a higher hindgut digesta WHC without gaining BW. Possibly, grass that is harvested early could resolve the contradiction of BW and forage intake for the equine athlete. The effects on exercise response and dehydration need further investigations. The small daily starch intake of <2 g/kg BW when the horses were fed CMGH seemed to influence lactate-utilising bacteria. EGH fulfilled the energy and protein requirements for exercising horses without adding concentrate, which may promote intestinal health.

## Figures and Tables

**Table 1:** Daily intake of DM, energy and dietary components of the diets ^1,2^ (in g/100 kg BW if not otherwise stated).

	CMGH	EGH	LH	SEM	*p*-Values
DM (kg/100 kg BW per day)	2.1 ^a^	1.8 ^b^	1.9 ^b^	0.06	0.007
Energy (MJ/100 kg BW per day) ^3^	18.6 ^a^	19.4 ^a^	16.2 ^b^	0.48	<0.001
Crude protein	298 ^b^	318 ^a^	250 ^c^	8.9	<0.001
Crude fibre	632 ^b^	526 ^c^	688 ^a^	18.9	<0.001
Neutral detergent fibre	1112 ^a^	966 ^b^	1032 ^b^	32.0	0.011
Acid detergent fibre	691 ^b^	569 ^c^	770 ^a^	21.0	<0.001
Acid detergent lignin	88 ^b^	55 ^c^	132 ^a^	4.0	<0.001
Hemicellulose ^4^	421 ^a^	398 ^a^	262 ^b^	14.0	<0.001
Cellulose ^4^	603 ^a^	513 ^b^	638 ^a^	18.1	0.001
Starch	157 ^a^	1 ^c^	9 ^b^	2.1	<0.001
Water soluble carbohydrates ^5^	56 ^a^	50 ^a^	19 ^b^	2.4	<0.001
Glucose	3 ^a^	4 ^a^	2 ^b^	0.5	0.007
Fructose	20 ^b^	31 ^a^	8 ^c^	2.1	<0.001
Sucrose	23 ^a^	11 ^b^	4 ^c^	0.5	<0.001
Fructans	3	3	2	0.6	0.694
Maltodextrins	6 ^a^	2 ^b^	3 ^b^	0.9	0.003
Calcium	21	24	21	1.1	0.110
Phosphorus	7 ^a^	6 ^a^	5 ^b^	0.2	<0.001
Magnesium	4 ^b^	4 ^b^	6 ^a^	0.2	<0.001
Sodium	5	5	5	0.2	0.120
Potassium	36 ^b^	50 ^a^	49 ^a^	1.5	<0.001

^1^ CMGH: concentrate (oats, soybean meal) and mature grass haylage (35:65 energy ratio), EGH: early and mature grass haylage (80:20) (+ small amount soybean meal), LH: lucerne haylage and mature grass haylage (80:20). ^2^ Least square means with the pooled SEM. ^3^ Forage ME values were calculated from the in vitro digestible organic matter values [27]. ^4^ Hemicellulose and cellulose concentrations were calculated by weight difference: NDF-ADF and ADF-ADL, respectively. ^5^ Free glucose, free fructose, sucrose, fructans and maltodextrins. ^a,b,c^ Within a row means without a common superscript letter differ (*p* < 0.05).

**Table 2:** Horses water intake by drinking and via the feed and water output via faeces and urine, after three weeks of adaptation to forage-based diets differing in fibre composition and maturity ^1,2^.

	CMGH	EGH	LH	SEM	*p*-Values
Water intake (kg/day)	24.6 ^a^	25.2 ^a^	22.4 ^b^	0.83	0.048
Water in feed (kg/day)	8.7 ^b^	6.3 ^c^	11.0 ^a^	0.30	<0.001
Total water intake ^3^ (kg/day)	33.3	31.5	33.4	1.03	0.255
Water in faeces (kg/day)	18.8 ^a^	13.1 ^b^	17.6 ^a^	1.00	0.001
Water in urine (kg/day)	7.6 ^b^	10.5 ^a^	7.9 ^b^	0.52	0.032
Total water output (kg/day)	26.3	23.6	25.5	1.02	0.096
Difference intake-output (kg/day)	7.0	8.0	7.9	0.27	0.055

^1^ Least square means with the pooled SEM, *n* 6. ^2^ CMGH: concentrate (oats, soybean meal) and mature grass haylage (35:65 energy ratio), EGH: early and mature grass haylage (80:20) (+ small amount soybean meal), LH: lucerne haylage and mature grass haylage (80:20). ^3^ Water intake by drinking + water in feed. ^a,b,c^ Within a row means without a common superscript letter differ (*p* < 0.05).

**Table 3:** Pearson correlations between horses’ daily total water intake and daily DM, NDF and ADF intake from the total diet ^1^ and from the forage part of the diet (*n* 6).

	Total Water Intake	
	Correlation Coefficient	*p*-Values
Total DM intake	0.714	<0.001
Total forage DM intake	0.522	0.026
Total NDF intake	0.704	0.001
Total forage NDF intake	0.730	<0.001
Total ADF intake	0.677	0.002
Total forage ADF intake	0.631	0.005

^1^ CMGH: concentrate (oats, soybean meal) and mature grass haylage (35:65 energy ratio), EGH: early and mature grass haylage (80:20) (+ small amount soybean meal), LH: lucerne haylage and mature grass haylage (80:20).

**Table 4:** Water-holding capacity (WHC) in g H_2_O/g dry faeces and DM in % of faeces, after three weeks of adaptation to forage-based diets differing in fibre composition and maturity ^1,2^.

	CMGH	EGH	LH	SEM	*p*-Values
WHC filtration method	9.9	10.7	9.4	0.41	0.142
WHC centrifugation method	11.1 ^b^	12.6 ^a^	11.4 ^b^	0.28	0.014
DM	18.6	19.0	18.7	0.65	0.781

^1^ Least square means with the pooled SEM, *n* 6. ^2^ CMGH: concentrate (oats, soybean meal) and mature grass haylage (35:65 energy ratio), EGH: early and mature grass haylage (80:20) (+ small amount soybean meal), LH: lucerne haylage and mature grass haylage (80:20). ^a,b^ Within a row means without a common superscript letter differ (*p* < 0.05).

**Table 5:** Microbial counts, SCFA, SCFA ratio ((acetate + butyrate)/propionate) and pH in faeces, after three weeks of adaptation to forage-based diets differing in fibre composition and maturity ^1,2^.

	CMGH	EGH	LH	SEM	*p*-Values
Microbial count (log CFU/mL)					
Total anaerobic bacteria	8.0	8.3	7.5	0.42	0.382
Pectinolytic bacteria	7.1	6.9	6.5	0.22	0.270
Xylanolytic bacteria	6.8	6.6	6.4	0.39	0.517
Cellulolytic bacteria	5.9	4.7	5.4	0.22	0.059
Amylolytic bacteria	5.3	5.1	5.5	0.26	0.647
Lactate-utilising bacteria	6.6 ^a^	5.8 ^b^	5.7 ^b^	0.18	0.036
SCFA (mmol/L)					
Total SCFA ^3^	41.4	51.5	40.0	4.67	0.314
Acetate	28.8	34.9	28.0	3.44	0.411
Propionate	8.4	11.4	8.1	0.95	0.171
Butyrate	2.0 ^b^	3.0 ^a^	1.6 ^b^	0.24	0.034
SCFA ratio	3.7	3.3	3.8	0.14	0.084
Valerate	0.3	0.5	0.3	0.04	0.072
Iso-butyrate	1.0	0.8	1.1	0.05	0.075
Iso-valerate	1.0	1.1	0.9	0.08	0.384
Total lactate ^4^	6.5	4.2	3.9	0.64	0.095
D-lactate	5.0	3.3	2.6	0.65	0.114
L-lactate	1.4	0.8	1.1	0.16	0.153
pH					
	6.9 ^a^	6.5 ^b^	7.2 ^a^	0.07	0.005

^1^ Least square means with the pooled SEM, *n* 6. ^2^ CMGH: concentrate (oats, soybean meal) and mature grass haylage (35:65 energy ratio), EGH: early and mature grass haylage (80:20) (+ small amount soybean meal), LH: lucerne haylage and mature grass haylage (80:20). ^3^ Acetate, propionate, butyrate, valerate, iso-butyrate, iso-valerate. ^4^ D and L lactate. ^a,b^ Within a row means without a common superscript letter differ (*p* < 0.05).

## Data Availability

Data are available by request to the authors.
